# A self‐adhesive microneedle patch with drug loading capability through swelling effect

**DOI:** 10.1002/btm2.10157

**Published:** 2020-02-29

**Authors:** Sharon W. T. Chew, Ankur H. Shah, Mengjia Zheng, Hao Chang, Christian Wiraja, Terry W. J. Steele, Chenjie Xu

**Affiliations:** ^1^ School of Chemical and Biomedical Engineering Nanyang Technological University Singapore; ^2^ NTU Institute for Health Technologies, Interdisciplinary Graduate School Nanyang Technological University Singapore; ^3^ School of Materials Science and Engineering Nanyang Technological University Singapore; ^4^ National Dental Centre of Singapore Singapore

**Keywords:** bioadhesive, dendrimer, microneedles, self‐adhesive, transdermal drug delivery

## Abstract

Microneedles (MNs) offer a rapid method of transdermal drug delivery through penetration of the stratum corneum. However, commercial translation has been limited by fabrication techniques unique to each drug. Herein, a broadly applicable platform is explored by drug‐loading via swelling effect of a hydrogel MN patch. A range of small molecule hydrophilic, hydrophobic, and biomacromolecule therapeutics demonstrate successful loading and burst release from hydrogel MNs fabricated from methacrylated hyaluronic acid (MeHA). The post‐fabrication drug loading process allows MeHA MN patches with drug loadings of 10 μg cm^−2^. Additional post‐fabrication processes are explored with dendrimer bioadhesives that increase work of adhesion, ensuring stable fixation on skin, and allow for additional drug loading strategies.

## 1 INTRODUCTION

Transdermal delivery via microneedle (MN) patches enhances skin penetration of therapeutics in a minimally invasive manner.^1^, ^2^, ^3^ It opens the possibility of pill‐free drug delivery of both small molecule and biomacromolecule therapeutics. Conventional drug loading strategies employ layer‐by‐layer,^4^ spray,^5^ or embedded coatings on the surface of MNs. The latter incorporates the drug directly into the MN matrix during fabrication.^6^, ^7^ However, surface coating is limited to the concentration of drugs that can be coated, and often requires the addition of excipients to improve the viscosity in order to achieve uniform coating.^8^ Pre‐loading of drugs into the MN matrix is constrained by the need of new fabrication procedures for different drugs.

Hydrogels as controlled‐release vehicles offer a variety of porous structures and chemical environments for tuning release kinetics and aqueous swelling.^9^, ^10^ Drugs can be incorporated either by gradients or pre‐mixing the hydrogel monomer(s) with therapeutics prior or during the fabrication.^9^ The tunable swelling property of hydrogels inspired us to develop poly(ethylene glycol) diacrylate (PEGDA)‐based hydrogel MNs for the delivery therapeutic peptides—which is especially important in prevention of scar tissues. For example, Gap 26 is a connexin mimetic peptide that inhibits cellular gap‐junction for the treatment and prevention of keloids.^11^ However, PEGDA MNs were restricted by swelling ratios below 20%. This leads to day long incubation period preventing therapeutic loading within minutes. To solve this issue, there is a need to devise a MN platform with high swelling ability for rapid drug loading by swelling effect.

**Figure 1:**
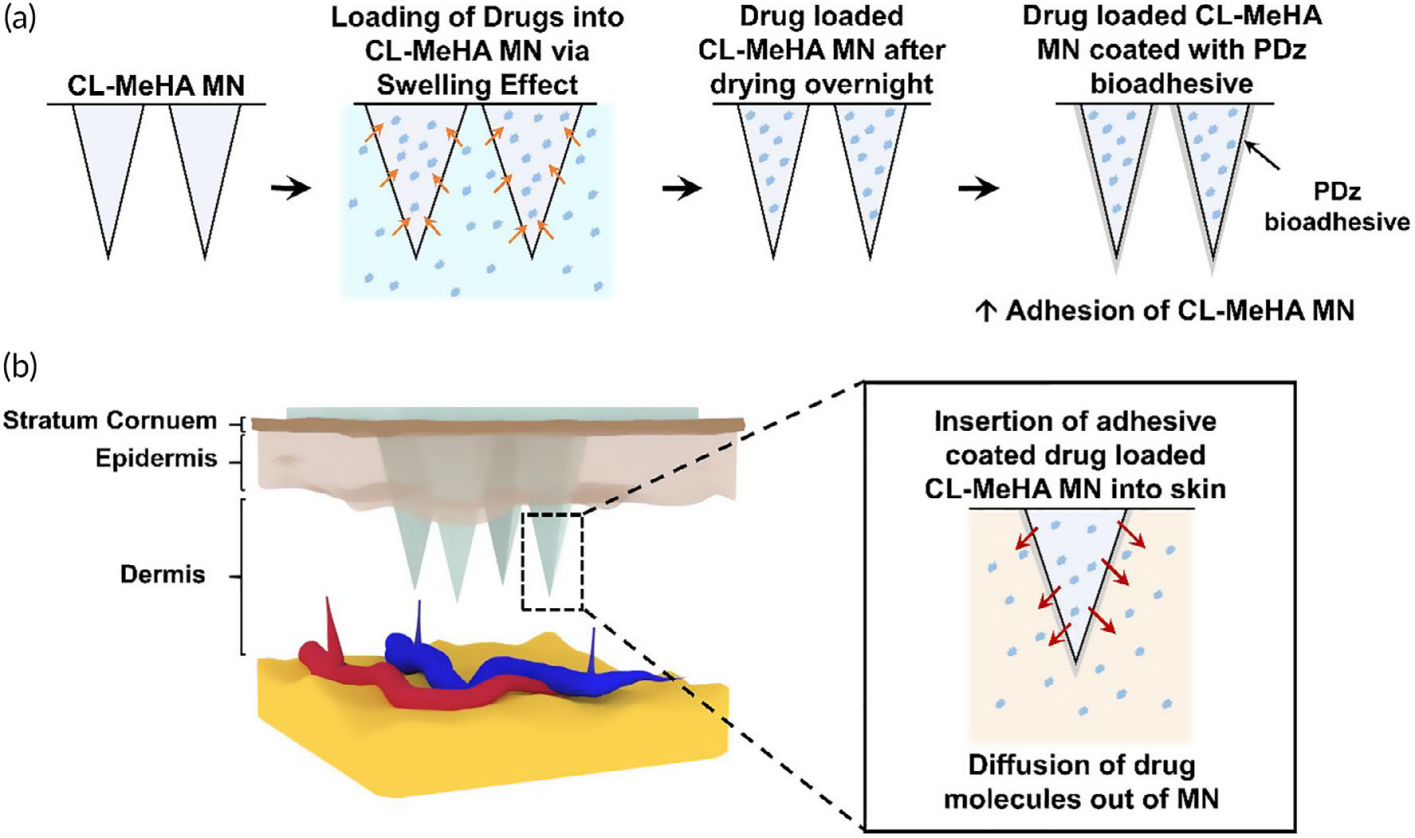
Schematic illustration showing (a) loading of drug solution into CL‐MeHA MN and adhesive coating of CL‐MeHA MN patches. (b) After loading of therapeutics into CL‐MeHA MN through swelling effect, the drug‐loaded CL‐MeHA MN patch can be directly administered onto the skin for drug delivery with improved adhesion. MeHA, methacrylated hyaluronic acid; MN, microneedle

Hyaluronic acid (HA) is known to hold up to 100 times its weight in water.^12^ Crosslinking can be exploited to control swelling. Recently, we demonstrated that methacrylated HA (MeHA)‐based hydrogel MNs could swell up to nine times its original weight in within minutes.^13^ The higher swelling ratio and shorter duration of MeHA MNs is hypothesized to exceed the transdermal drug delivery capabilities of PEGDA MNs. As a proof‐of‐concept, MeHA MNs are loaded with a range of molecules of various sizes, including fluorescein, fluorescein isothiocyanate (FITC)‐Dextran, and doxorubicin hydrochloride (Dox) through swelling effect (Figure 1). These molecules represent small hydrophilic molecules (<500 Da), large hydrophilic molecules (>500 Da), and hydrophobic small molecules. The swelling degree of MeHA MN patch is controlled through tuning the crosslinking degree of MeHA, which influence the drug loading capability. MeHA MN patch also demonstrates good peel adhesion strength (~0.20 N cm^−1^) against porcine ear skin, which is approximately two‐fold higher in comparison with medical‐grade adhesive—Tegaderm™ film. Furthermore, MeHA MN patches are coated with non‐aqueous photocuring tissue adhesive to improve fixation up to 40% (uncoated MeHA MN patches) via UVA (365 nm) mediated covalent crosslinking.^14^, ^15^, ^16^ Thus, the MeHA MN patch eliminates the need for adhesive films for fixation during application, reducing the possibility of skin irritations such as local inflammation (e.g., redness) or erythema that is observed with usage of commercial adhesives.^17^, ^18^ Additionally, it may offer an additional platform for long term drug loading and biosensing.

## 2 RESULTS

### 2.1 Development and characterization of swellable MeHA MNs

MeHA polymer was synthesized by modifying HA with methacrylate anhydride according to the protocol reported previously.^13^ The degree of methacrylation was 70.5% according to ^1^H NMR map (Figure S1). The MNs were fabricated from MeHA polymer using template molding approach.^13^ Briefly, MeHA solution was cast into the polydimethylsiloxane (PDMS) negative mold replicated from stainless‐steel master template (height: 1000 μm; base width: 300 μm) and dried naturally before exposure to ultraviolet (UV) illumination for crosslinking. The resulting patch was a 10 × 10 MN array on an 8 mm × 8 mm supporting base patch. The needle retained its pyramidal shape with a height of ~800 μm and base of ~250 μm (Figures S2a and S2b). The slight decrease in the dimension of MeHA MN patch in comparison to the master template was due to the shrinkage of the PDMS negative mold during the replicate process.

### 2.2 Five‐minute UV exposures yield reproducible and intact MN structures

MeHA MNs were reported to swell rapidly in minutes within aqueous solutions and this is tunable through controlling the UV exposure time.^13^ Longer UV exposure time would result in smaller swelling ratio, thereby decreasing the drug loading capacity. We examined the swelling behavior of MeHA MNs crosslinked with 3, 4, 5, 10, and 15 min of UV exposure (namely CL3, CL4, CL5, CL10, and CL15, respectively) by measuring the mass change before and after incubation in 1X phosphate buffered saline (PBS). As shown in Figure S3a, all CL‐MeHA MNs fully swelled within 1 min, with CL3‐MeHA MN giving the highest swelling ratio of ~5.96 and CL15‐MeHA MN having a swelling ratio of ~1.87. The size of all MeHA MN patches increased during the 30‐min incubation (Figure S3b). After removal from the PBS solution, the patches dried naturally and returned to their original dimensions.

Although CL3‐MeHA MN exhibited the highest swelling ratio in the experiment, it became soft and brittle. Missing MN tips were also observed after removal from the PBS solution for both CL3 and CL4‐MeHA MN patches (Figure S3). Hence, CL5‐MeHA MN is the best choice going forward, given their relatively high swelling ratio (~2.74) and stability in the swelling process.

### 2.3 CL5‐MeHA MNs resist swelling in organic solvents, but have tunable swelling in aqueous solutions

Besides PBS, we also explored the swelling of CL5‐MeHA MNs in solvents with a range of relative polarities (RP). Chloroform (RP = 0.259), acetone (RP = 0.355), N,N‐dimethylformamide (DMF) (RP = 0.386), dimethyl sulfoxide (DMSO) (RP = 0.444), ethanol (RP = 0.654), and methanol (RP = 0.762) are chosen as they span a wide range of polarities.^19^ No significant swelling effect of CL5‐MeHA MNs was observed after 3‐hr incubation (Figure S4a). However, the patch did swell in 70% ethanol and Dulbecco's Modified Eagle's Medium (DMEM). This suggested that MeHA MNs may be limited to aqueous solvents, indicating that drugs of hydrophilic nature are more suitable for loading into MeHA MN via the swelling effect. Finally, SEM examination confirmed that MeHA MNs were stable, regardless of the polarities (Figure S4b).

### 2.4 CL5‐MeHA MNs demonstrates a wide range of drug loading via swelling effect

**Figure 2:**
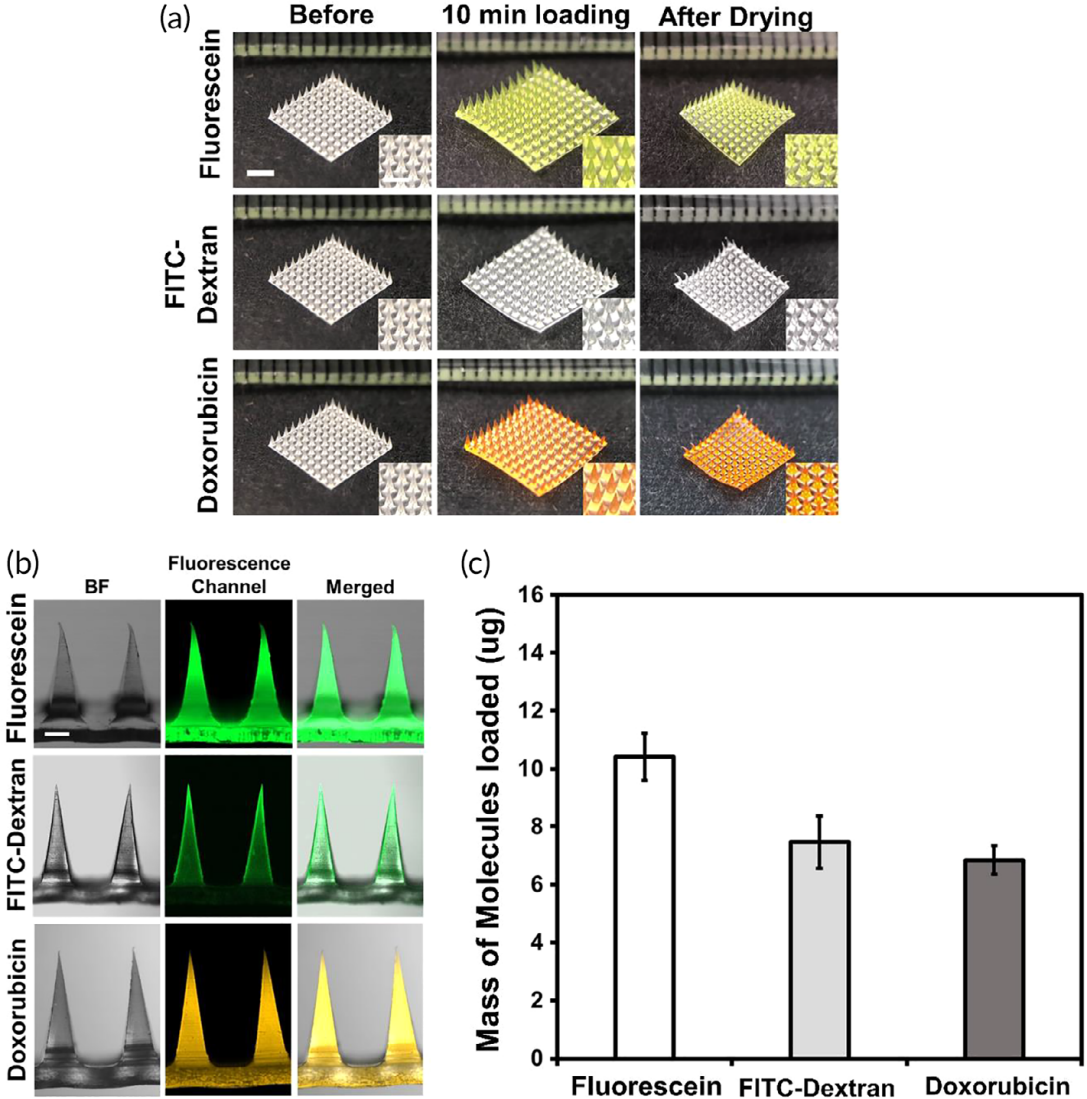
Loading of molecules into CL5‐MeHA MN patches via swelling effect. (a) Representative images of CL5‐MeHA MN patches before and after the loading of fluorescein, FITC‐Dextran, and Doxorubicin. Scale bar: 2 mm. (b) Representative fluorescence images of CL5‐MeHA MN loaded with various molecules. Scale bar: 200 μm. (c) The quantification of mass of molecules loaded in CL5‐MeHA MN patches. FITC, fluorescein isothiocyanate; MeHA, methacrylated hyaluronic acid; MN, microneedle

Next, the drug loading capabilities of CL5‐MeHA MNs were examined using fluorescein, FITC‐Dextran, and Dox. They represent three groups of molecules of different molecular weights, that is, small hydrophilic molecules (<500 Da), large hydrophilic molecules (> 500 Da), and hydrophobic small molecules. As shown in Figure 2, CL5‐MeHA MNs are able to incorporate the molecules of different sizes—with mass loading of up to 10.4 μg ± 0.8 for fluorescein; 7.5 μg ± 0.9 for FITC‐Dextran; and 6.8 μg ± 0.5 for Dox, per MN patch. MN patches reached loading saturation within 10‐min incubation under isotonic conditions. This meets one of our objectives in rapid drug loading (Figure S5).

The molecular sizes have influence over the amount that can be loaded through swelling effect. The higher fluorescence intensity observed in Figure 2b show that higher amount of fluorescein is loaded into the MN patch. On the other hand, the amount of Dox loaded was lower (6.8 μg ± 0.5) due to its relatively lower solubility in aqueous solvents (solubility in water: 100 mg ml^−1^ for fluorescein and 2.6 mg ml^−1^ for Dox), 20 requiring it to be dissolved in small amount of DMSO prior to dilution with aqueous solvents. In addition, the loading content of Dox is much lower compared with that of FITC‐Dextran despite the smaller molecular size of Dox. This can be attributed to the higher hydrophobicity of Dox as demonstrated by the higher octanol/water partition coefficients, log K_o/w_, of the Dox at 1.27 compared to that of FITC‐Dextran and fluorescein (−0.773 and −0.615, respectively).^20^, ^21^ The higher hydrophobicity of Dox may impede its diffusion into the polymer matrix of the MN patches with high water affinity. Therefore, hydrophobicity is a vital factor in determining the loading amount of therapeutics into the MN patch apart from molecular size.

### 2.5 Mechanical property and drug release profile of CL5‐MeHA MN patches

**Figure 3:**
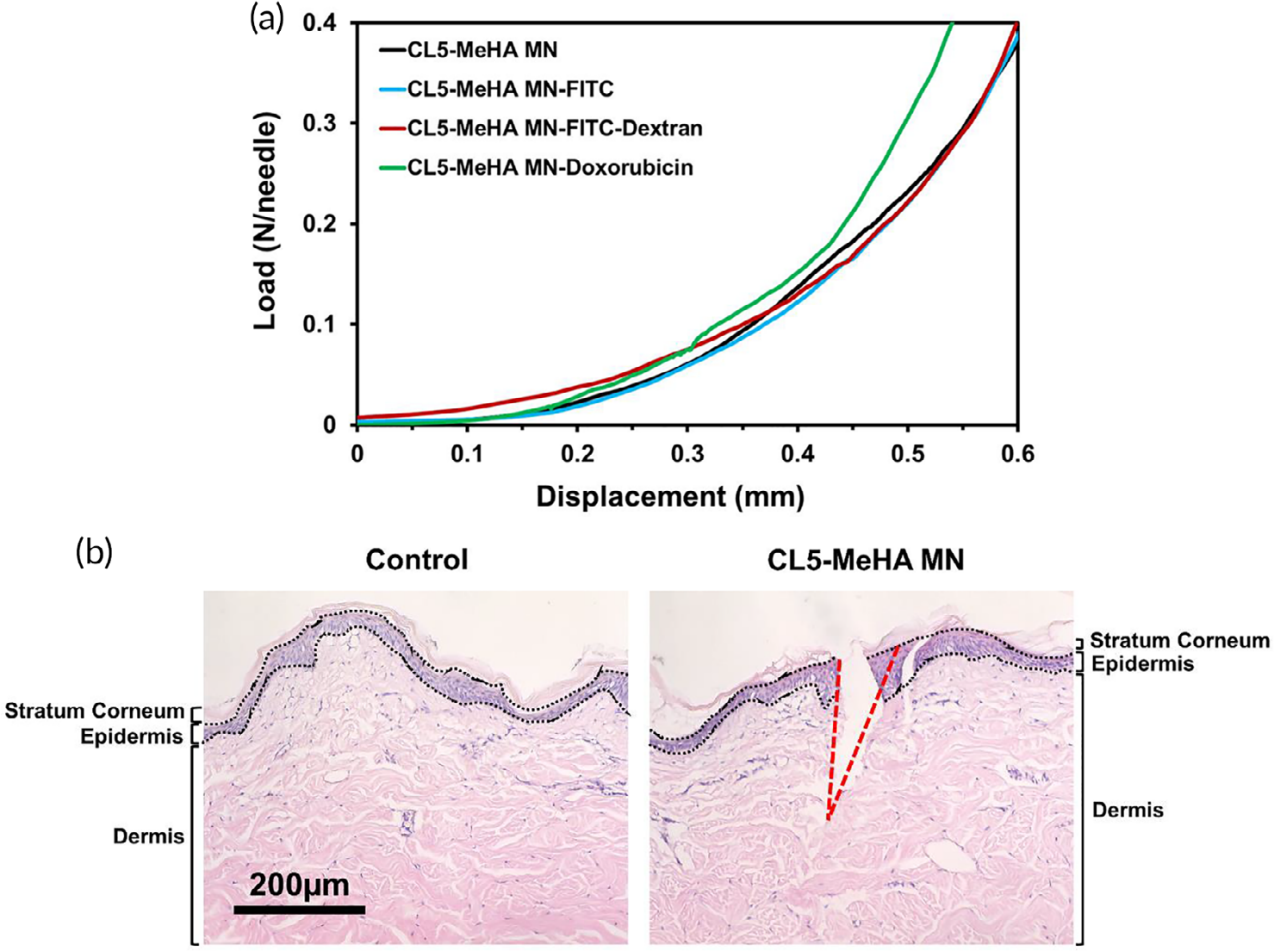
Mechanical properties of CL5‐MeHA MNs. (a) The force versus displacement curves of crosslinked MeHA MNs loaded with or without the three molecules (i.e., fluorescein, FITC‐Dextran, and Doxorubicin). (b) H&E‐stained cross‐section image of porcine ear skin that was punched with CL5‐MeHA MNs. The skin layers are demarcated by the black dotted line and labeled. The region where MN insertion took place is outlined by the red dotted line. Scale bar: 200 μm. FITC, fluorescein isothiocyanate; MeHA, methacrylated hyaluronic acid; MN, microneedle

We assessed the mechanical strength of CL5‐MeHA MNs after drug loading using compression test (Figure 3a). All patches gave similar force versus displacement profiles except Dox‐loaded CL5‐MeHA showing slightly steeper profile. This shows that the loading process did not have any impact on the mechanical property of the MN patches. None of the MN patches broke under the compressive force of 0.4 N per needle, which is sufficient for penetration into the skin, considering the minimum penetration force required for normal skin penetration to be 0.058 N per needle.^22^, ^23^

The skin penetration ability of CL5‐MeHA MNs was subsequently confirmed in a porcine ear skin model, which serves as an in vitro model for human skin.^24^ CL5‐MeHA MNs were applied into the porcine ear skin with a pressure of 150 g cm^−2^ (approximately 1.5 N). Histology study demonstrated successful disruption of stratum corneum (~25 μm thickness) (Figure 3b).^25^ The penetration depth was approximately 600 μm for the 800 μm CL5‐MeHA MNs. Elastic nature of skin prevents 100% MN insertion due to deformation of porcine skin during MN insertion.^26^ CL5‐MeHA MN patches also penetrate across viable epidermis (50–120 μm) and reach the superficial dermis. This allows the released drug to diffuse and reach the capillary blood supply in the dermal layer for circulation in the body.^27^

**Figure 4:**
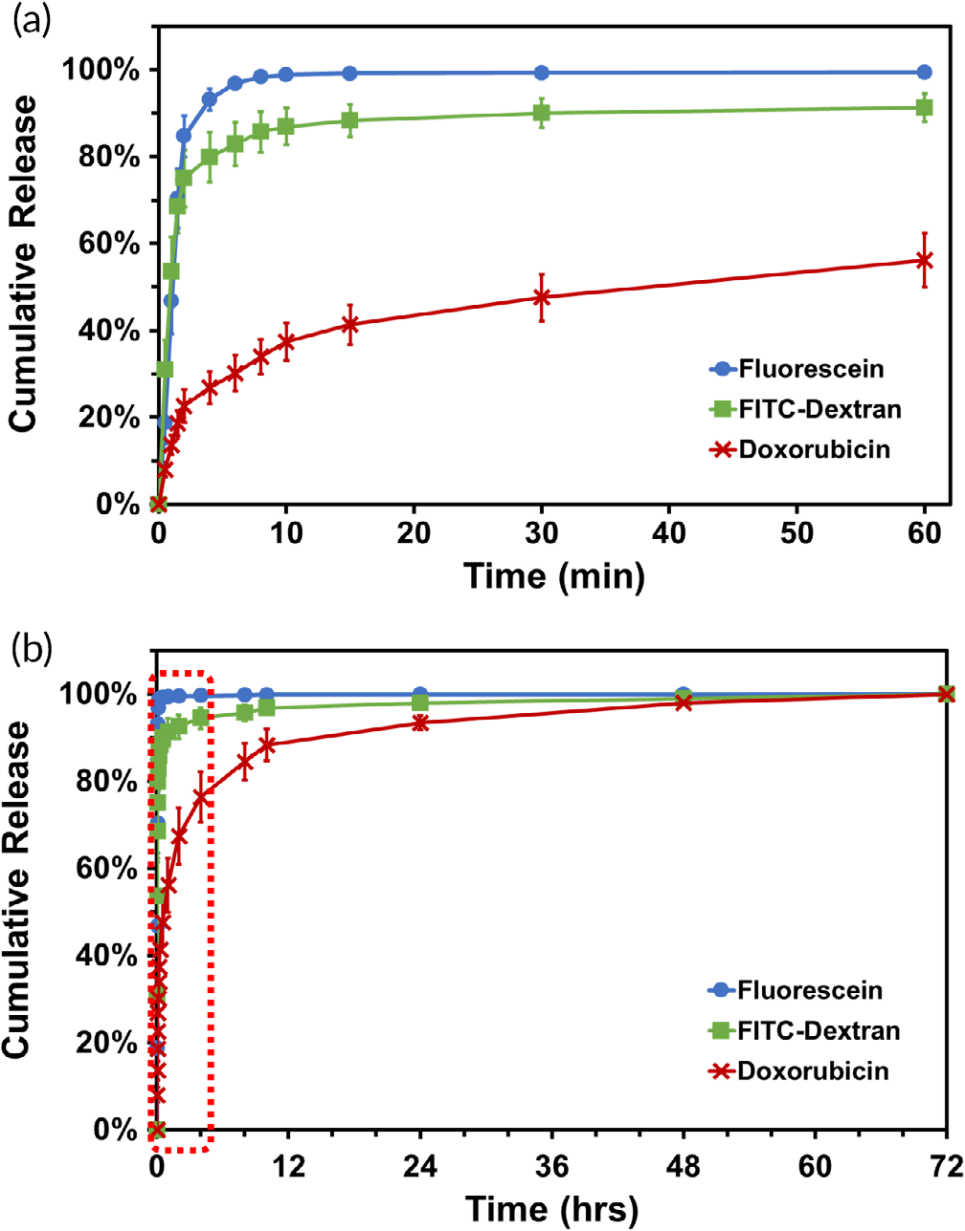
Release profiles of fluorescein, FITC‐Dextran, and Doxorubicin from CL5‐MeHA MN patches over (a) the first 1 hr and (b) 72 hr. FITC, fluorescein isothiocyanate; MeHA, methacrylated hyaluronic acid; MN, microneedle

We examined the release profiles of molecules from the CL5‐MeHA MN patches after incubation in 1X PBS at 37°C to mimic physiological conditions. The release kinetics was assessed until >90% release of the molecules was recorded from the MN patches. As depicted in Figure 4a, >80% of fluorescein, FITC‐Dextran and >50% of Dox were released from the MN patches within 30 min. The high water‐affinity of MeHA allows for the exchange of molecules in the polymer matrix of CL‐MeHA MNs resulting in release of higher amount of hydrophilic molecules into the PBS in a short duration. Slower release kinetics observed in Dox‐loaded MN patches could be due to the poor hydrophilicity nature of the drug that also resulted in lower amount loaded into the patches through swelling effect. Over 95% of the molecules were released after 72 hr (Figure 4b).

### 2.6 Adhesion strength of CL5‐MeHA MNs

**Figure 5:**
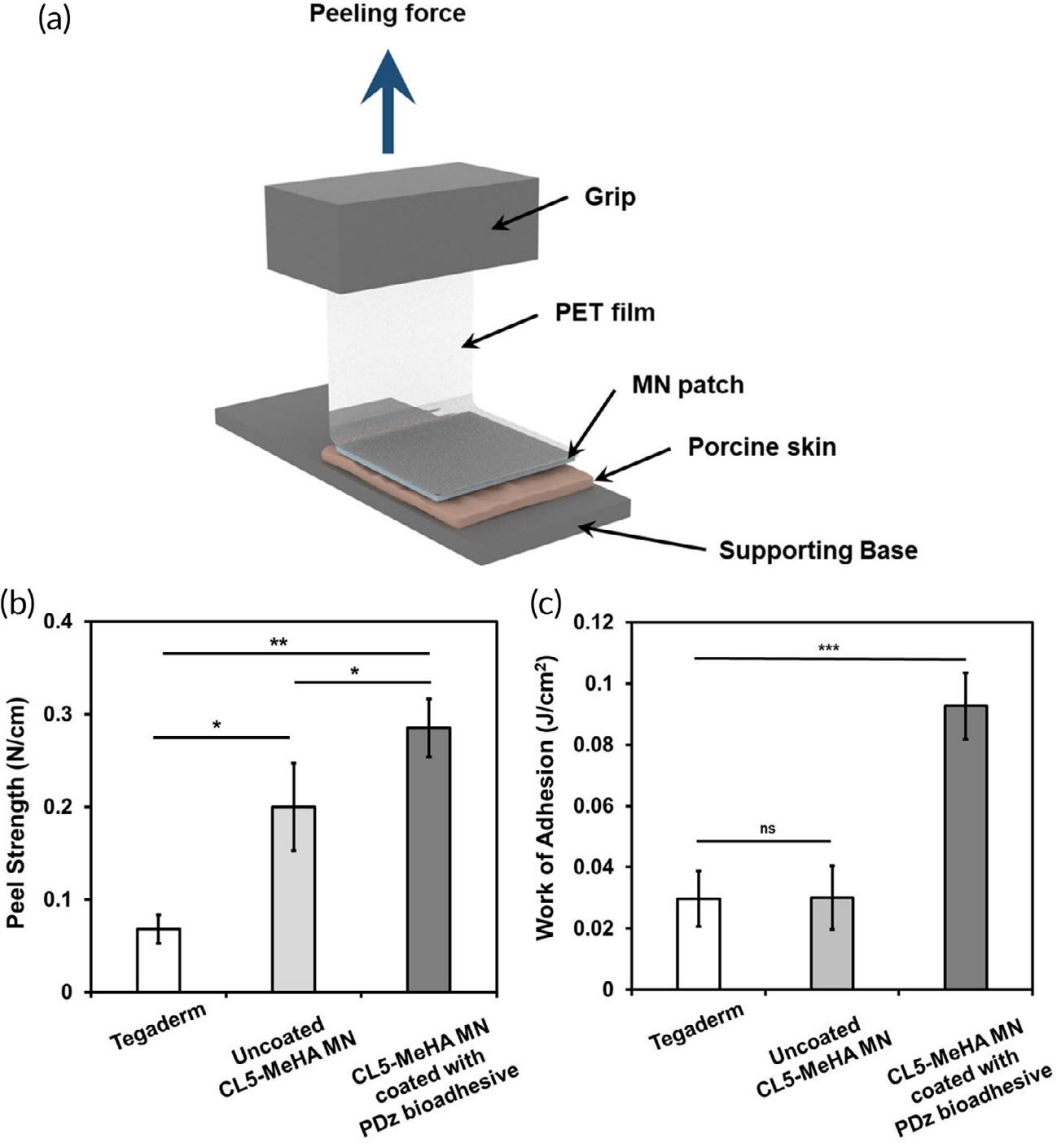
Skin adhesion of CL5‐MeHA MN patch. (a) Schematic illustration of the Peel test. (b) Peel strength of CL5‐MeHA MN patches by 90° peeling test. (c) Work of adhesion of CL5‐MeHA MN patches by 90° Peel test. MeHA, methacrylated hyaluronic acid; MN, microneedle

Finally, we assessed the adhesive strength of CL5‐MeHA MN patches with the 90° peeling test on porcine ear skin (Figure 5a).^24^, ^28^ CL5‐MeHA MN patches showed a maximum peel strength of 0.20 N cm^−1^ ± 0.047, which is approximately two times higher than that of the Tegaderm™ film (0.068 N cm^−1^ ± 0.015) (Figure 5b). As the surface area of MN patches is larger as compared to that of the Tegaderm™ film, this contributed to higher effective contact area between the MeHA MN patches and skin tissue, thus improving the peel adhesion strength of the MN patch on rough surface contours such as skin.

To further enhance the peel adhesion strength for high friction or wet tissue environments (e.g., foot soles and perspiration), the surface of the MN patch was coated with PAMAM‐g‐diazirine (PDz) tissue adhesives that impart contact sterility and conformal wetting.^14^, ^16^ These tissue adhesives were chosen because of their liquid material properties and on‐demand adhesive activation. The low viscosity adhesive will not interfere with the MN skin penetration and can be activated after MN insertion through the UVA transparent MeHA MN. Upon UVA stimulation (365 nm), PDz bioadhesives show rapid gelation within 30 s and can provide lap‐shear adhesion strengths up to 250 kPa in wet environment.^14^, ^16^ As discussed earlier, MeHA MN patches only swell in the presence of water, so PDz bioadhesives were dissolved in liquid PEG 400, which was shown to have minimal swelling in ethanol/alcohol environments.^14^, ^16^, ^29^ With PDz bioadhesive, the formation of carbene crosslinking between the bioadhesive layer and skin tissue upon UV stimulation increased the interfacial bonding, thus improving the adhesion strength of the adhesive coated MN patch. The thickness of the PDz bioadhesive coating was 40 μm ± 4.5 and the adhesion strength of the MN patch was 0.29 N cm^−1^ ± 0.032 (Figure 5b), 40% increment compared with uncoated MeHA MNs. Figure 5c further demonstrated that the coating of PDz bioadhesive onto MN patch improves the adhesion strength significantly, as threefold increase in energy is required to break the bonding between the PDz bioadhesive‐coated MN patch and skin tissue. (0.093 J cm^−2^ ± 0.011). This can allow for stable fixation and longer period application on the skin.

## 3 DISCUSSION

Post‐processed MeHA MN patches are demonstrated for the first time as drug carrier for a wide range of therapeutics. MeHA MNs are highly swellable in aqueous solutions (Figure S4), which allowed the efficient and effective loading of molecules of various sizes (fluorescein, FITC‐Dextran, and Dox) through swelling effect (Figures 2 and S5). The swelling ability of MeHA MN was tunable by controlling the UV crosslinking duration (Figure S3), which in turn influenced the drug loading capability. The drug‐loaded MeHA MNs were mechanically robust to penetrate the stratum corneum, allowing drug released to access the epidermis and dermal layers. The high water‐affinity of MeHA allowed the patch to swell in skin, which permitted the drug release into the skin tissue and may be responsible for the observed adhesion strength.

MeHA MNs exhibit adhesion strength that is better than the medical‐grade adhesive—Tegaderm™ film. This could be due to the soft mechanical property of the patch upon swelling might contribute to the delaying of the crack propagation in the peeling region through energy dissipation. The rapid swelling of MeHA MN in the skin may result in opposing force that is strong enough to cause delamination of the MN patches from the skin after a period of application. This was addressed by coating non‐aqueous photocuring tissue adhesive (PDz bioadhesive) on the MeHA MN patch to improve the adhesion strength.

The key issue with the current MN platforms is the lack of post‐processing methods available for drug loading after MN fabrication. Thus, every formulation will require unique fabrication and production lines, overall impeding clinical translation. Thus far to the best of our knowledge, there is no single MN platform that can load various therapeutic classes. The MeHA MN patch introduced in this study leverages the superior hydrophilicity of MeHA polymer for drug loading by employing green chemistry principles and avoid VOCs. The post‐processing requires approximately 24 hr (10 min for loading of therapeutics by swelling effect and overnight drying of the MN patch in ambience temperature) and this duration can be further decrease by exploring different drying processes (e.g., lyophilization and vacuum drying) in the future studies. Hence, this MeHA MN platform demonstrates excellent potential for translation as off‐the‐shelf medical product given its versatility for loading different therapeutics and the relatively short post‐processing duration. Furthermore, the improved adhesion peel strength allows for stable fixation of the MN patch onto the skin for longer duration application of the MN patch without the need for additional medical adhesives. The coating of non‐aqueous PDz bioadhesive also allows for incorporation of therapeutics in the adhesive layer. Release kinetics of therapeutics from PDz bioadhesive can be tuned by varying the dosage of UV‐A, which can be pursue in future study for tunable release kinetics. To load protein drugs, UV activation of the bioadhesive may affect the bioactivity of certain protein‐based therapeutics; therefore, future study can be conducted to explore the use of other instant curing adhesives (e.g., electrocuring adhesives and thermocuring adhesives) for coating on the MN patch. In addition to drug delivery, previous study has demonstrated that MeHA MNs can also extract skin interstitial fluid (ISF) for biosensing application.^13^ Combination of drug delivery and extraction of ISF can be explored in future studies for the development of a theranostic transdermal delivery device using MeHA MN.

## 4 CONCLUSION

There is an unmet need to develop a MN platform that can incorporate various therapeutics post‐MN fabrication process in a facile manner. Herein, a self‐adhesive MN platform with universal drug loading capability is developed to address this need. The excellent swelling ability of MeHA MN allows for drug loading via swelling effect in a short duration (~10 min) and contributes to the good adhesion strength. The adhesion strength of the MN platform can be enhanced with a coating of non‐aqueous photocuring tissue adhesive, which allows for incorporation of therapeutics. Future study on the therapeutic efficacy in animal models can be explored to demonstrate the clinical potentials of this platform.

## 5 EXPERIMENTAL SECTION

### 5.1 Synthesis of MeHA polymer

MeHA polymer was synthesized according to the reported protocol.^13^ Briefly 4.0 g of 300 kDa HA was dissolved in 200 ml deionized water (DI water) and left under continuous stirring overnight. Subsequently, the solution was transferred to a 4°C environment and 133 ml DMF and 4.8 ml methacrylic anhydride were then added to the solution respectively, in a dropwise manner. The pH of the solution was controlled between pH 8–9 by adding 1 M NaOH whilst keeping the reaction in 4°C environment under continuous stirring for another 18 hr. Next, 9.88 g sodium chloride (NaCl) was added to the solution to achieve 0.5 M NaCl before precipitation in 100% ethanol. The precipitate was washed with ethanol three times again prior to dissolving in DI water. The solution was dialyzed against DI water over a seven‐day period and the purified polymer (MeHA) was obtained through lyophilization. Characterization of the polymer was performed using ^1^H NMR spectroscopy (Bruker Avance II 300 MHz NMR) and the degree of polymerization was determined by digital integration of the anomeric protons signals or methyl protons signals of HA and of the methacrylate proton signals at ~6.1, ~5.7, and ~1.9 ppm.

### 5.2 Fabrication of CL‐MeHA MN patches

PDMS negative mold was prepared by pouring PDMS (10:1 w/w ratio of pre‐polymer base to curing agent) over the stainless‐steel MN master structure (Micropoint Inc, Singapore), followed by degassing in vacuum oven for 10 min and curing at 70°C for 1 hr. MeHA polymer (50 mg ml^−1^) and photo‐initiator (Irgacure 2959, 0.5 mg ml^−1^) were dissolved in DI water. The solution was then casted into the PDMS negative mold and centrifuged at 4,000 rpm for 5 min to fill the needle cavities. Additional solution was then added to ensure a robust supporting base of the MN patch. The MN patches were then left to dry overnight in fume hood. After drying, the MeHA MN patches were gently removed from the mold and exposed to UV light (wavelength = 360 nm, intensity = 17.0 mW cm^−2^, model 30, OAI) for a determined duration (3, 5, 10, and 15 min) for crosslinking. The crosslinked MeHA MN patches with UV exposure for 0, 3, 5, 10, and 15 min were named as MeHA MN, CL3‐MeHA MN, CL5‐MeHA MN, CL10‐MeHA MN, and CL15 MeHA‐MN, respectively.

### 5.3 Swelling behavior of CL‐MeHA MNs in PBS

The swelling ratio of CL‐MeHA MNs was obtained by comparing their mass before and after incubation in 1X PBS. Firstly, the dry mass (W_o_) of CL‐MeHA MN of various crosslinking degree (CL3, CL5, CL10, and CL15) was recorded before any treatment. Then the MN patches were directly immersed in 1X PBS solution. At each determined timepoint, the MN patch was removed from PBS and any excessive water was removed by tissue paper and the mass (*W*_t_) was measured immediately. The swelling ratio was calculated using the equation of W_o_ W_t_^−1^.

### 5.4 Swelling abilities of CL5‐MeHA MNs in solvents with different polarities

CL5‐MeHA MNs were directly immersed in solvents—chloroform, acetone, DMSO, DMF, methanol, 70% ethanol, 99.9% ethanol, and DMEM for 3 hr. Then MN patches were taken out and excess solution was removed by tissue paper. The morphology of CL5‐MeHA MNs before and after incubation was observed under SEM and optical images were taken using smartphone fixed with macro lens. For optical imaging and processing of the MN patches, the MN patch was placed against a black background next to a centimeter scale ruler. A 20X macro lens fixed onto the camera of a smartphone was used to capture image of the MN patch within 15 mm length of the ruler. The scale bar was then constructed based on the centimeter scale ruler.

### 5.5 Loading of CL5‐MeHA MN patches through swelling effect

Aqueous solutions of fluorescein, FITC‐Dextran (3–5 kDa), and Dox hydrochloride were prepared with 1X PBS and diluted to a concentration of 100 μg ml^−1^. CL5‐MeHA MNs were immersed in these solutions (500 μl) for a determined duration. Subsequently, at each determined timepoint, CL5‐MeHA MN was taken out and excess solution was removed from the surface by tissue paper before drying in fume hood. The mass of the MN patches before and immediately after incubation in the solution was measured and recorded. The fluorescence intensity of the solution after incubation with MN patches was then measured using microplate reader (Synergy H4) (fluorescein/FITC‐Dextran: excitation 495 nm/emission 519 nm; Dox hydrochloride: excitation 519 nm/emission 595 nm), and the concentration was quantified using a calibration curve of the solution of each molecule with a known concentration (0–200 μg ml^−1^). The amount of molecules loaded into the patches was then calculated using the following equation.

$$\text{Amount of molecules loaded},M_{\text{drug}}=\left(C_{0}\times \text{Volume}\right)-C_{1}\times \left(\text{Volume}-\frac{∆M}{\rho }\right)$$

where *C*_o_ is the concentration of the solution prior to incubation with MN patches (100 μg ml^−1^), ΔM is the difference in mass of the MN patch before and after loading, *C*_1_ is the concentration of the solution after incubation with MN patches, and ρ is the density of water.

The CL5‐MeHA MNs loaded with the three molecules were then imaged using confocal laser scanning microscope (Carl Zeiss, LSM 710) with excitation of 488 nm laser and emission at 493–634 nm.

### 5.6 Mechanical property and ex vivo porcine ear skin insertion test

The mechanical strength of drug‐loaded CL5‐MeHA MNs was assessed using Instron 5543 Tensile Meter. An axial force was applied on a flat‐headed stainless‐steel probe at a constant speed of 0.5 mm min^−1^ toward CL5‐MeHA MN that was placed with its tips facing upward on the flat aluminum plate. The displacement was measured until a pre‐set maximum force of 0.4 N per tip was achieved. To evaluate the skin penetration efficiency, CL5‐MeHA MNs were applied onto porcine ear skin by thumb force for 1 min. The skin tissue was then embedded in FSC22 Frozen Media (Leica Microsystem) and cryosections (10 μm thick) were prepared using a cryostat (CM1950, Leica Microsystems). The skin sections were then stained with hematoxylin and eosin (H&E) for histological analysis.

### 5.7 In vitro release from CL5‐MeHA MNs

CL5‐MeHA MN loaded with the molecules were immersed in 1X PBS and kept at 37°C. At the desired timepoints, 50 μl of the solution was transferred to a 96‐well plate and the fluorescence intensity was examined with microplate reader (Synergy H4). 50μl of fresh PBS was added back to the solution and the concentrations were calculated based on the standard curves and the cumulative release of drugs were calculated for the different timepoints for the different molecules.

### 5.8 Peel adhesion test

The 90° peel test was conducted against porcine ear skin to evaluate the peel strength of the CL5‐MeHA MN patches using the tensile tester with a 50 N load cell. (Chatillon Force Measurement Products) The porcine ear skin was secured to a platform and the MN patches was applied on the porcine ear skin by thumb force for 1 min. The supporting base of MN patches was attached onto a PET film (thickness: 0.02 mm) using cyanoacrylate adhesive, which was secured by the grip of the tensile tester. The MN patches were peeled at 90° from the porcine ear skin at speed of 3 mm min^−1^. The peel strength (N cm^−1^) was calculated by measuring the maximum load (N) of the peel test and dividing by the width of the MN patches (0.8 cm). For the peel test of bioadhesive‐coated CL5‐MeHA MN patches, after application by thumb force, the MN patches was exposed to UV light (wavelength = 365 nm, intensity = 200 mW cm^−2^ for 90 s) for curing of the bioadhesive prior to the peel test. For control, commercially used adhesive—Tegaderm™ film was applied on the porcine ear skin for 1 min before the peel test. The tegaderm™ film was cut into 0.8 cm by 0.8 cm to be consistent with the MN patches dimension before the peel test. The peeling tests were carried out in triplicates.

### 5.9 Preparation of PDz bioadhesive

Polyamidoamine‐g‐diazirine 15% grafted (PDz) was synthesized according to the reported protocol.^15^ Set amount of PDz, PEG 400, and tertiary PEGs (10 kDa; 5% w/w in MeOH) were suspended in a 2 ml Eppendorf and diluted with methanol to obtain a homogeneous solution. The adhesive was formulated to have the following weight ratios PDz (30%): PEG 400 (60%): PEG 10K (10%) as this formulation has shown to high storage modulus (~270 kPa) with well‐defined porous structure.^14^ Before use, the MeOH suspended adhesive was evaporated (vacuum oven at 37°C for 96 hr) to obtain a pale yellow viscous liquid and stored in dark at 4°C.

### 5.10 Thickness measurement of PDz bioadhesive coating on CL5‐MeHA MNs

The thickness of the adhesive coating was derived from the mass of the CL‐MeHA MN patches before and after the coating. Firstly, the dry mass of the CL‐MeHA MN was recorded before the coating process. Subsequently the CL‐MeHA MN patches was coated with 100 μl of the PDz adhesive at left to dry in the vacuum oven at 37°C for 2 hr. Then the mass of the coated CL‐MeHA MN patches was recorded. The thickness of the adhesive was calculated using the following equation:

$$\text{Thickness of coating}=\frac{\Delta M}{\rho }\times \frac{1}{\mathrm{SA}}$$

where Δ*M* is the difference in mass of the MN patch before and after coating, ρ is the density of adhesive at 0.997 g cm^−3^ and SA is the surface area of the MN patch at 1.61 cm^2^.

### 5.11 Statistical analysis

All data were obtained from at least three independent experiments with at least three parallel samples per condition in each experiment and were expressed as means ± *SDs*. Significance was determined by Student's *t*‐test. A probability value of *p* < .05 was considered significant (*p* ≤ .01).

## 6 CONFLICT OF INTERESTS

The authors declare no conflict of interest.

## Supporting information


**Figure S1** ^1^H NMR spectra of MeHA polymer.
**Figure S2. a)** SEM and **b)** optical image of one crosslinked MeHA MN patch. Scale bars are 500 μm and 1,000 μm respectively.
**Figure S3. a)** The swelling behavior of CL‐MeHA MN patches crosslinked with different UV exposure times in a 30‐minute period. **b)** Images of CL‐MeHA MN patches in the swelling process. Scale bars: 2 mm.
**Figure S4. a)** Images of CL5‐MeHA MN patches before and after the 3‐hour incubation in solvents with different polarities. Inserts are the zoom‐in images of the MN patches. Scale bar: 2 mm. **b)** SEM images of CL5‐MeHA MNs after the 3‐hour incubation in solvents with different polarities. Scale bar: 20 μm.
**Figure S5. a)** Optimization of loading duration of CL5‐MeHA MN patches in the various solutions. **b)** Images of CL5‐MeHA MN patches in the loading process. Inserts show close‐up of needles. Scale bar: 2000 μm.
