# Supplementary material for: A self‐adhesive microneedle patch with drug loading capability through swelling effect
Source: Bioeng Transl Med. 2020 Feb 29;5(2):e10157. doi: 10.1002/btm2.10157 (PMC7237146; doi:10.1002/btm2.10157)
Supplement: Supplementary file 1 — Figure S1 1H NMR spectra of MeHA polymer. Figure S2. a) SEM and b) optical image of one crosslinked MeHA MN patch. Scale bars are 500 μm and 1,000 μm respectively. Figure S3. a) The swelling behavior of CL‐MeHA MN patches crosslinked with different UV exposure times in a 30‐minute period. b) Images of CL‐MeHA MN patches in the swelling process. Scale bars: 2 mm. Figure S4. a) Images of CL5‐MeHA MN patches before and after the 3‐hour incubation in solvents with different polarities. Inserts are the zoom‐in images of the MN patches. Scale bar: 2 mm. b) SEM images of CL5‐MeHA MNs after the 3‐hour incubation in solvents with different polarities. Scale bar: 20 μm. Figure S5. a) Optimization of loading duration of CL5‐MeHA MN patches in the various solutions. b) Images of CL5‐MeHA MN patches in the loading process. Inserts show close‐up of needles. Scale bar: 2000 μm. [file BTM2-5-e10157-s001.docx]

Supporting Information

A Self-adhesive Microneedle Patch with Drug loading Capability through Swelling Effect

Sharon W. T. Chew, Ankur Harish Shah**,** Mengjia Zheng, Hao Chang, Christian Wiraja, Terry W. J. Steele, Chenjie Xu*


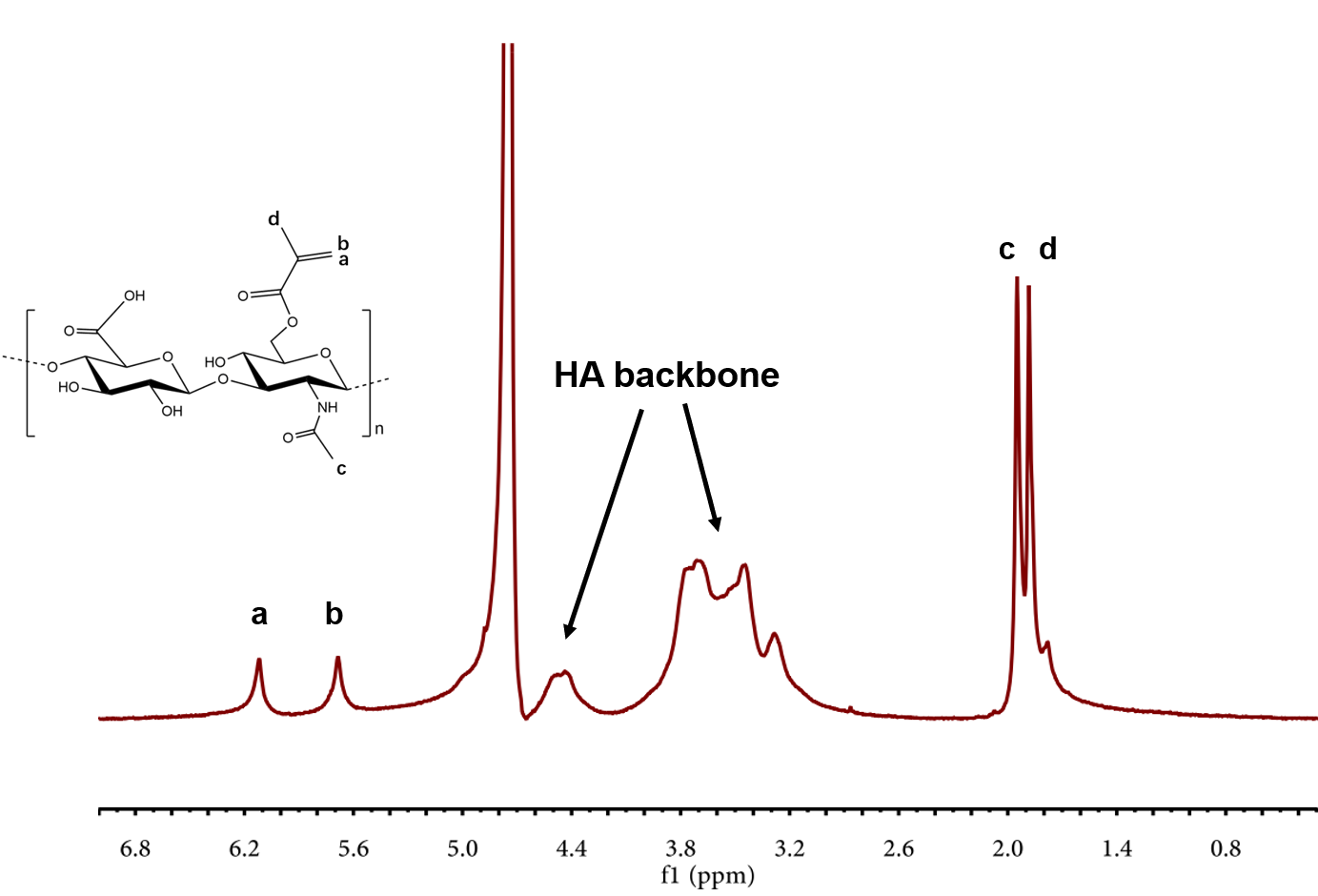


**Figure S1.** ^1^H NMR spectra of MeHA polymer.


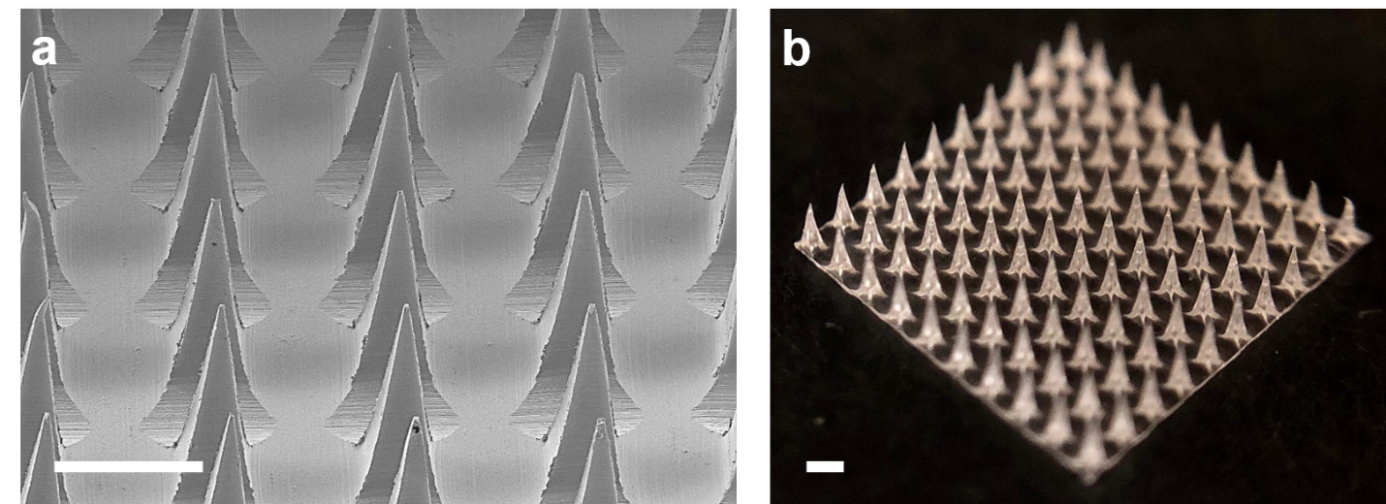


**Figure S2. a)** SEM and **b)** optical image of one crosslinked MeHA MN patch. Scale bars are 500 µm and 1000 µm respectively.


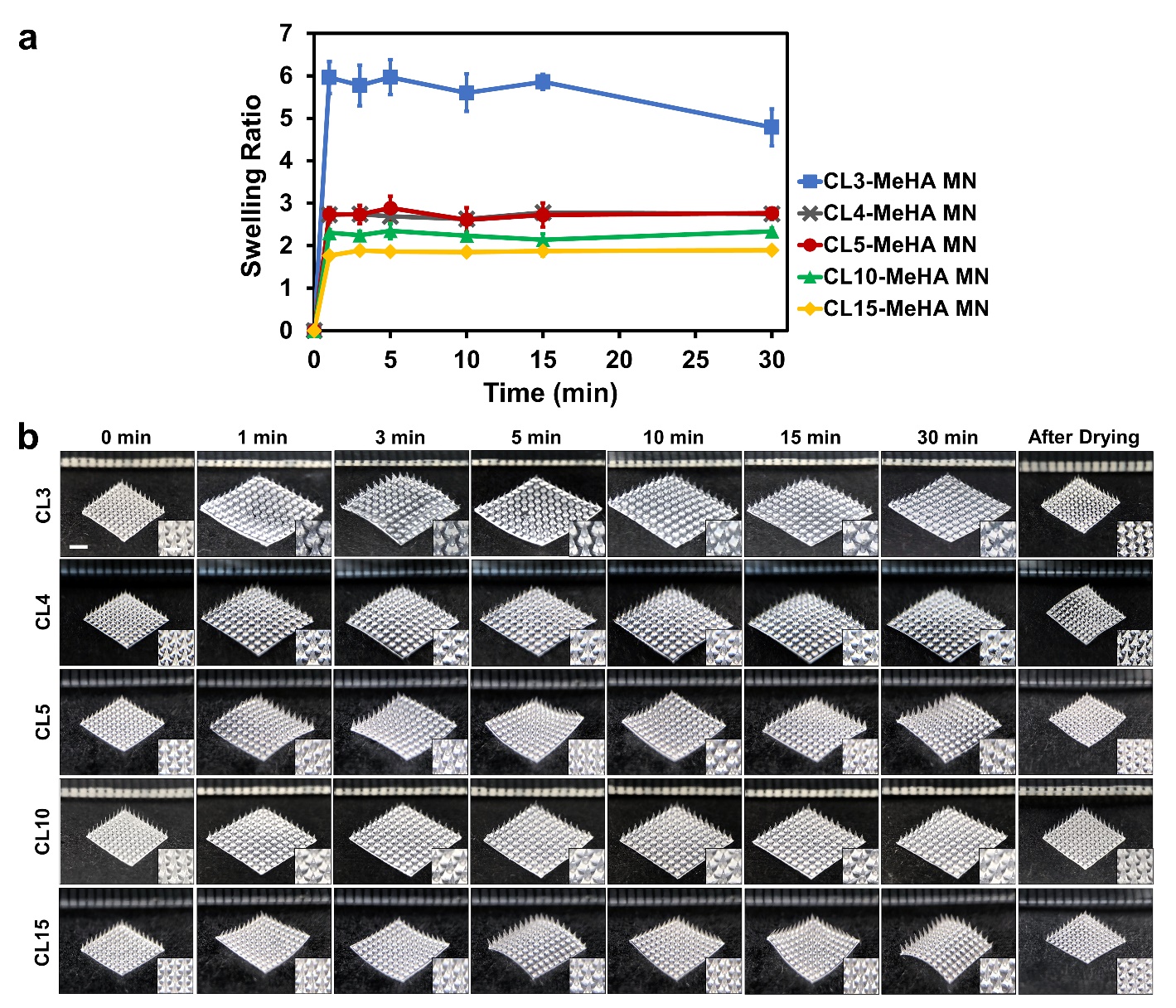


**Figure S3. a)** The swelling behavior of CL-MeHA MN patches crosslinked with different UV exposure times in a 30-minute period. **b)** Images of CL-MeHA MN patches in the swelling process. Scale bars: 2 mm.


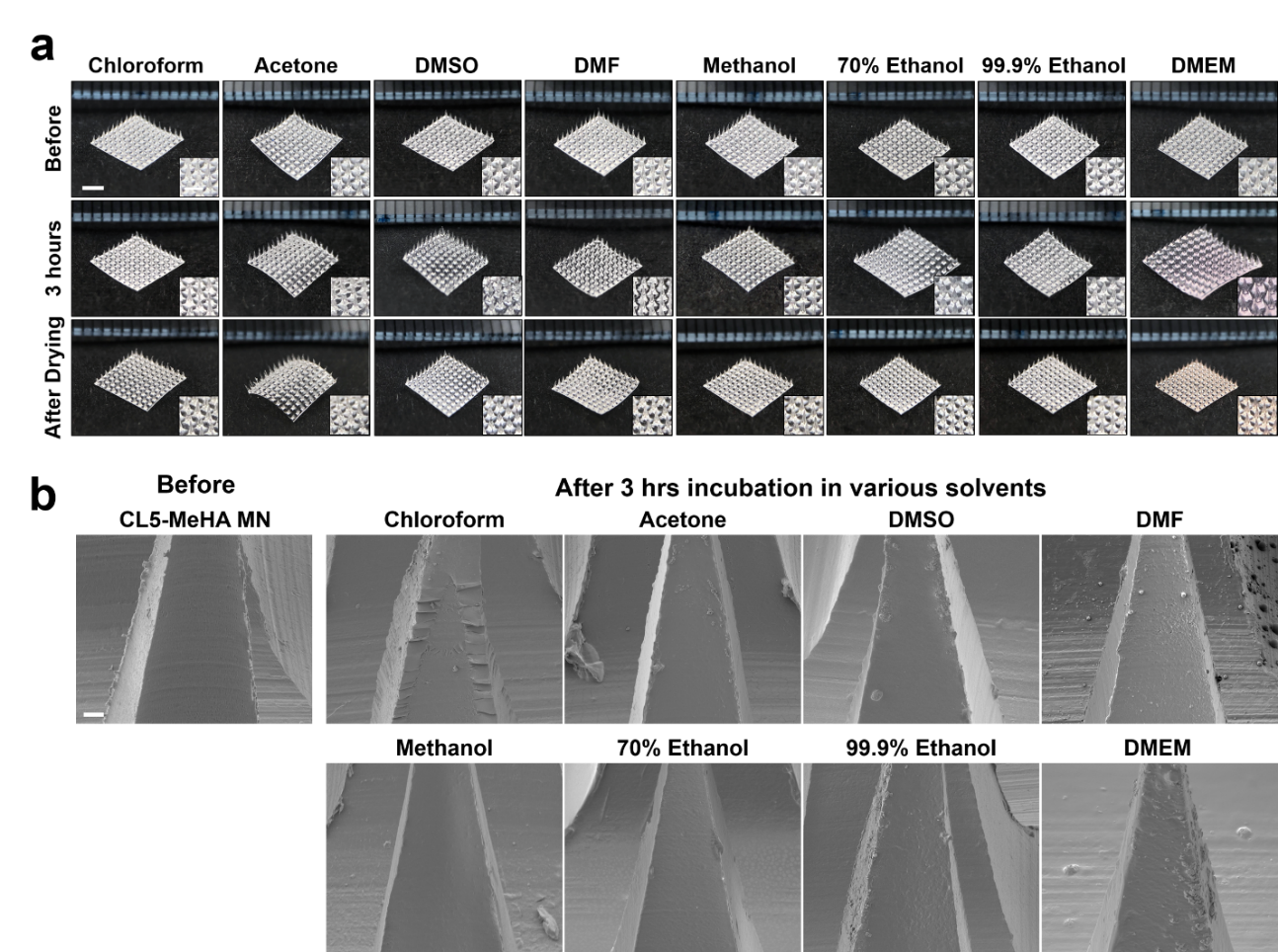


**Figure S4. a)** Images of CL5-MeHA MN patches before and after the 3-hour incubation in solvents with different polarities. Inserts are the zoom-in images of the MN patches. Scale bar: 2 mm. **b)** SEM images of CL5-MeHA MNs after the 3-hour incubation in solvents with different polarities. Scale bar: 20 µm.


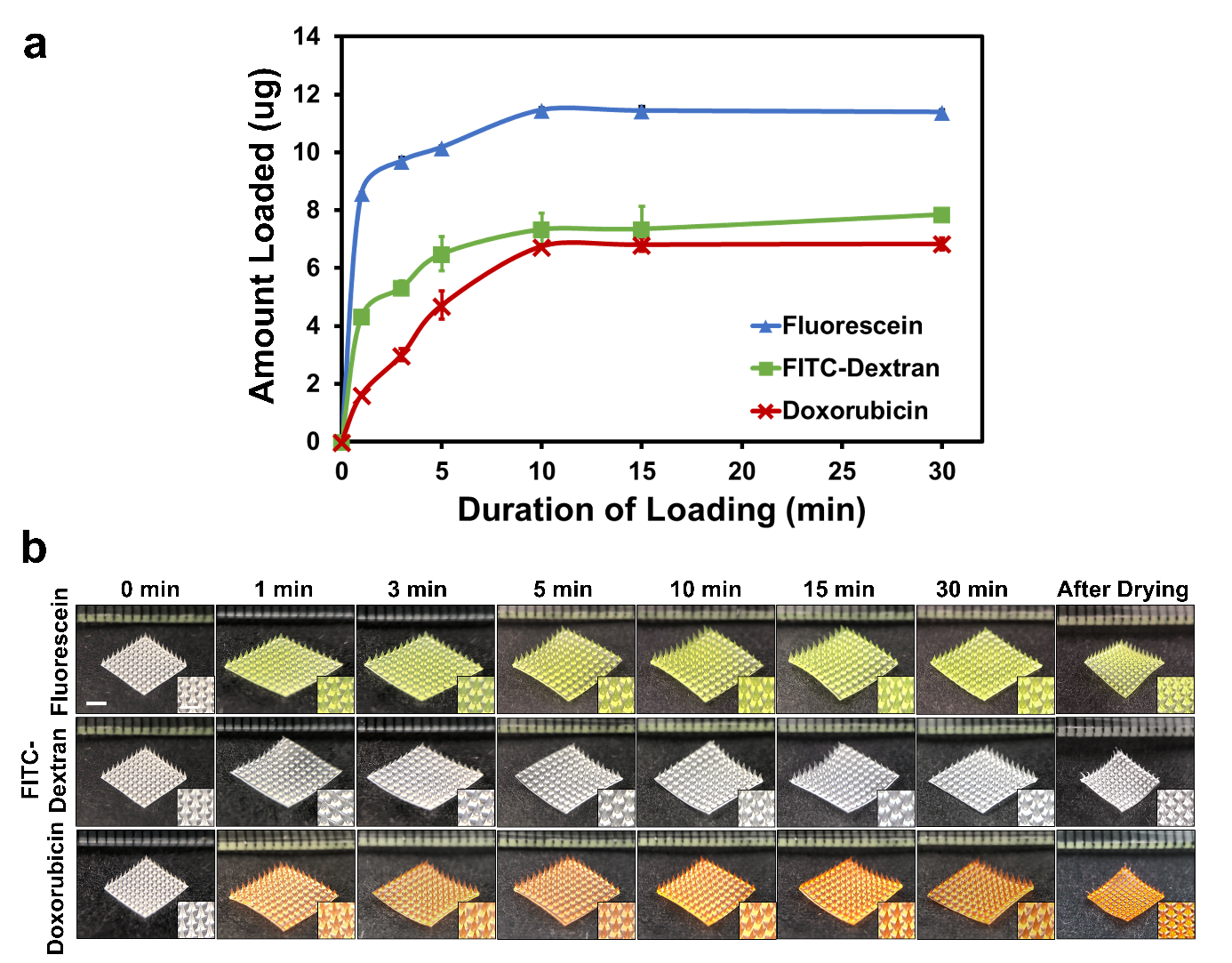


**Figure S5. a)** Optimization of loading duration of CL5-MeHA MN patches in the various solutions. **b)** Images of CL5-MeHA MN patches in the loading process. Inserts show close-up of needles. Scale bar: 2000 µm.
